# The effect of preheating of composite resin on its color stability after immersion in tea and coffee solutions: An *in-vitro* study

**DOI:** 10.4317/jced.56438

**Published:** 2019-12-01

**Authors:** Farideh Darabi, Ali Seyed-Monir, Sanaz Mihandoust, Dina Maleki

**Affiliations:** 1 Department of Operative Dentistry, Dentistry Faculty, Guilan University of Medical Sciences, Rasht, Guilan, Iran; 2 Doctor of Dental Surgery; 3 Dental Student, Student Research Committee, Dentistry Faculty, Guilan University of Medical Sciences, Rasht, Guilan, Iran

## Abstract

**Background:**

One of the concerns in using composite resins is color change. The aim of this study was to investigate the effect of preheating on color stability of composite resins when immersed in coffee and tea.

**Material and Methods:**

This experimental study included 60 composite disks. The samples were divided into 2 groups, one group prepared at room temperature and the other prepared at 68 °C. After curing, the samples were placed in 37 °C distilled water for 24 hours. The color of the samples was measured (t0) using spectrophotometer according to CIE-L*a*b* system. The samples of each group were then divided into 3 subgroups and respectively immersed in distillated water, coffee and tea for 30 days and the final color (t1) was measured. The difference between the measured colors was calculated (∆E) and the results were analyzed using version 21.0 of SPSS software, Paired t-test, ANOVA, Tukey’s test, and Dunnett t-test.

**Results:**

The preheated composites showed significantly lower staining in the coffee solution than the room temperature composites (*p*<0.0001). In contrast, no statistically significant difference was observed for the tea solution (*p* =0.317). The staining of the preheated composites in distillated water was higher than those in the room temperature, however, the difference was not significant (*p* =0.99).

**Conclusions:**

Within the limits of this study, preheating was effective to improve color stability of composite resin after long time immersion in coffee solution.

** Key words:**Composite resin, color stability, preheating.

## Introduction

Composite resins are one of the most common dentistry materials because of their ability to band with enamel and dentin, similarity with tooth structure in term of color and mechanical properties, ease of clinical application and comparatively low cost (1). Although in recent years the quality of composite resin restorations has been improved, the discoloration of these materials compromises the success of such restorations over the long term (2-5).

The discoloration of the composites is multifactorial and can have endogenous, exogenous, and/or idiopathic origin. The endogenous factors are related to the structure of the material such as the chemical change of the matrix, the degree of polymerization, and amount, size and distribution of the filler particles. On the other hand, exogenous factors are responsible for the absorption of pigmentation from external sources that are related to oral hygiene, smoking, and nutrition (6,7).

The degree of composite polymerization affects the chemical stability of the material and is directly related to its color stability (7,8). The unconverted dual carbon bands not only predispose the material to destructive reactions that results in the reduction of color stability and release some chemicals such as formaldehyde and methacrylic acid, but also facilitate the penetration of solvents from mouth environment to the polymeric network leading to the destruction of newly formed chains (9).

One technique that has been used and studied for the improvement of physical and mechanical properties of resin materials is preheating of them before their contact with teeth and exposure to light (7,8). In this method, before placing in a cavity, the material is heated to 50 to 68°C. This preheating of the composite leads to increase in molecular movement, higher polymerization degree, reduction of free monomers and increase in material flow (8). The increase of polymerization degree can improve the resistance of the material to discoloration by the reduction in water absorption following drinking colored beverages (7,10). Since the introduction of this method, the study on it is still ongoing and there are several instruments from different brands for heating the composites (11).

Despite some assumptions there are few studies on the effects of preheating on color stability of composite resins. Some studies reported the positive effects of such a technique (8,12) and one study did not find any effects (7). Therefore, the aim of this study was to evaluate the effect of preheating of a nano hybrid composite resin on the color stability when exposed to tea and coffee solution. The null hypothesis tested was that the preheating had no effect on the color stability of the materials, regardless of the coloring solutions.

## Material and Methods

In this experimental study, a nano hybrid composite resin (Herculite XRV Ultra, A2 shade) was selected. The properties of the studied composite resin are described in ( Table 1).

**Table 1:**
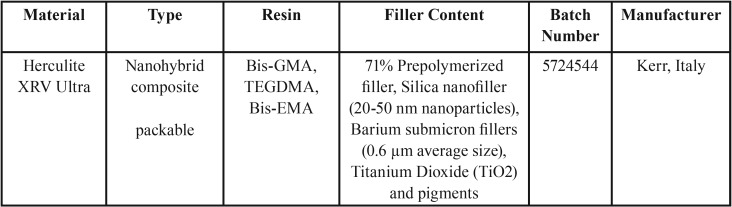
The properties of the studied composite resin.

60 disk samples with a diameter of 8 mm and thickness of 2 mm were prepared using a plastic mold. Specimens were divided into 2 groups according to the temperature of preparation (n=30).

Group 1 was prepared based on following instructions. The composite that was previously removed from the refrigerator and placed at room temperature (25°C) for at least 10 minutes was placed inside a plastic mold. After the initial straightening of the surface by a condenser, two celluloid strips were placed in the lower and upper parts of the samples in order to prevent the adhesion of the composite to glass slides. Then, the composite was placed between the slides and squeezed with hand pressure to the extent allowed by the plastic molds in order to allow the release of additional composite and obtain a sample with a flat surface and without bubbles. The samples were polymerized from both sides for 40 s using an LED light curing unit (Bluedent LED Smart, Bulgaria) at 1365 mW/cm2. The light intensity was monitored using a radiometer. The room temperature was maintained at 25°C using a cooling equipment and controlled by a mercury thermometer.

To prepare group 2, a portion of the composite was placed in a compule and then it was inserted in the compule gun. In the next step, the compule was placed in a heating instrument (Calset, AdDent Inc®, Danbury, CT, USA). After heating at 68°C, the composite was immediately inserted in the plastic mold and then it was cured from both sides as that of group 1. To reduce heat dissipation, the maximum time between the removing of the composite from the heater and placing it in the cavity was 10 seconds.

All samples were unmolded and placed in 37° C distillated water for 24 h until the polymerization was completed. The upper surface of the composites was polished for 20 s using aluminum oxide disks [Soflex (3M)]. Each disk was used for one sample. The color assessment of the samples was performed using a VITA Easy shade compact spectrophotometer. The samples were previously dried with moisture absorbent paper and were placed against a flat white background and then their colors were measured (t0).

The samples of each group were randomly divided into 3 subgroups (n=10). Samples of subgroup 1,2 and 3 were respectively immersed in distilled water, coffee and tea with a piece of thread in a vertical position, ( Table 2).

**Table 2:**
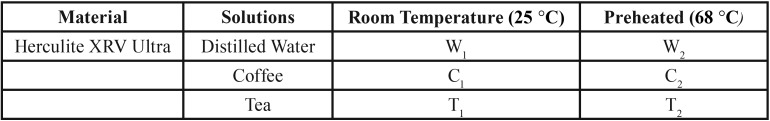
Division of Samples.

To prepare the coloring solutions of coffee, 3.6 gr of coffee powder was dissolved in 300 mL of boiling water and it was boiling for 10 minutes, for the tea solution, two tea bags were put in 300 mL of boiled water for 3 minutes. After that the samples were immersed in the solutions and placed in an incubator at 37°C for 30 days. The solutions were daily changed and samples were washed and brushed for one minute in order to remove any debris. The average time for drinking of a cup of coffee and the amount of drink have been reported about 15 minutes and 2-3 cups per day respectively (13). Therefore, an exposure time of 30 days seems to simulate an approximation of 30 months of coffee consumption. At the end of the 30-day period, all samples were placed against a flat white background and their color was measured (t1).

Color evaluation was performed using CIE-L*a*b*system (Commission International

**Figure 1:**
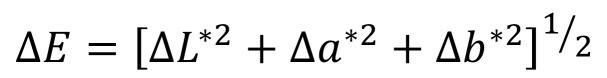
Formula.

De I’Eclairage) (14,15). The values of L* (lightness), a* (green-red axis), and b* (yellow-blue axis) were determined in each color reading. The color change (∆E) was calculated as follow: (Fig. 1).

The color change (∆E) ≥ 3.3, has been reported clinically visualized for human eyes and unacceptable (12).

Statistical analysis was performed using version 21.0 of SPSS software, Paired t-test, ANOVA, Tukey’s test and Dunnett t-test.

## Results

This experimental study was conducted on 60 samples of composite resin to evaluate the efficacy of preheating on its color stability after immersion in distilled water, coffee and tea.

**Figure 2:**
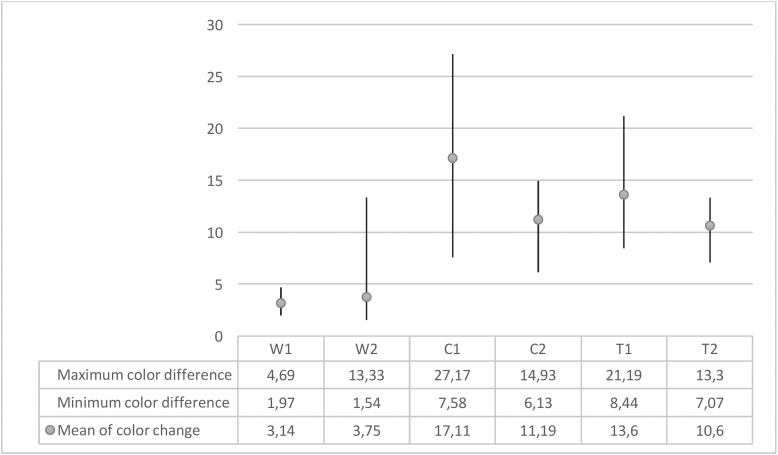
The mean of color change according to the temperatures and solutions.

The results showed a statistically significant difference in the mean color changes between the groups (*p*<0.0001). The highest and lowest color change were obtained for C1 (room temperature materials in coffee solution) and W1 (room temperature materials in distilled water) groups, respectively (Fig. 2).

 Table 3 indicates the paired comparison of ∆E between the groups. No significant difference was observed between the W1 and W2 groups (*p*=0.998). In contrast, the mean ∆E was significantly different between the C1 and C2 groups (*p*=0.002). Although ∆E was lower in the T2 than T1 group, the difference was not statistically significant (*p*=0.317).

**Table 3:**
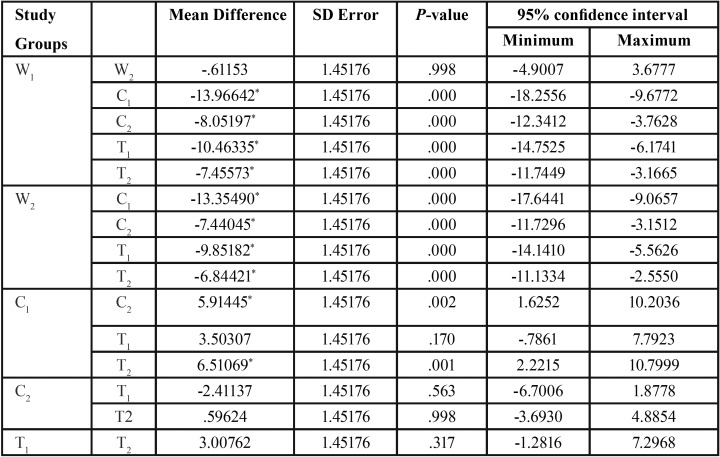
The paired comparison of color difference (∆E) between the studied groups.

## Discussion

The null hypothesis tested in this study was partially rejected as the preheated materials showed significantly lower discoloration when immersed in coffee but this reduction wasn’t significant in tea solution.

Discoloration of composite resin can be external and/or internal by adsorption and/or absorption of the colorant agents. Coffee and tea solutions have yellow pigments with different polarity. Previous studies have shown that preheating of composite resin rises its degree of polymerization and higher polymerization can cause higher resistance to discoloration (12,16,17). In our study preheating of composite resins caused higher resistance to discoloration of them in coffee than tea solution. Some authors declared that the color change of the composite in the coffee solution may be because of both adsorption and absorption of the yellow pigments. Preheating of the composites reduces absorption and penetration of the colorant solution via increase of polymerization degree and because of this, discoloration of preheated composite in coffee solution significantly reduced. On the other hand, tea has pigments which adsorbed on the material’s surface (13). Thus, an increase in the degree of polymerization has had less effect in the reduction of color change caused by tea.

In our study, in order to have more precision the color coordinates of samples were directly measured and analyzed by the spectrophotometer device (VITA Easy shade compact).

In previous studies preheating to 37, 50, 54, 55, 60 or 68°C were applied (17). However, in this study, maximum studied temperature (68°C) was selected to compensate heat dissipation.

In our study all composite disks after storage time in staining solutions, showed detectable staining (∆E ≥ 3.3) and the highest staining was observed for the coffee solution. This finding is in line with other studies (18,19). In short immersion time, coffee cause less discoloration than tea because of higher polarity and delayed release of pigments (13). Immersion time in this study was too long (30 days) which simulate approximately 30 months tea or coffee consumption (13).

The efficient polymerization and increased degree of conversion can affect the color stability of composite resins (20). This is because remained monomers cause the penetration of liquids such as colorant solutions to the polymeric network and also lead to hydrolytic destruction of newly formed chains that may results in the formation of color compounds (7).

In the present study, the preheating of the composites at 68°C significantly reduced the color change of them after 30 days immersion in coffee solution. Likewise, the staining of the composites was reduced in the tea solution, however, the difference was not significant. All composite disks after storage time in staining solutions, showed visible discoloration (∆E ≥ 3.3) and the highest discoloration was observed for the coffee solution. These findings are in accordance with other studies (8,12).

Sousa *et al.* (12), reported significant reduction in staining of preheated composite at 68°C after seven days immersion in cola and grape juice solutions. Similar findings are reported by Borges *et al.* (8). In contrast, Mundim *et al.* (7) did not observed significant difference in the color stability of preheated composites at 60°C in comparison with those at 8 and 25 °C. In the study of Mundim *et al.*, the specimens were placed in an accelerator system for 384 h while in the present study composite resins were immersed in coffee and tea solutions for 30 days.

In this study, we observed color change of the composites in the distillated water (control group), however, it was not statistically significant. Similar to this finding was reported by other studies (20-22). It seems water sorption itself and departure of soluble material can be cause of this discoloration. In addition, in the present study, the color change of the preheated composites was higher than room temperature ones, however, the difference was not significant. Because of the limited number of relevant studies, it is not possible to compare our finding and the justification of this observation requires more investigations.

One of the limitations of the present study was that the color of composites was not measured after re-polishing (after immersion in the coloring solutions). According to Zajkani *et al.* (13), the color change of composite resins immersed in coffee and tea solutions were significantly improved after re-polishing. In addition, in the clinic, due to the washing action of saliva and the oral hygiene of the patient, the color change of the restorative materials is less (12,20). Therefore, clinical investigations are required to assess the color stability of preheated composite resins when they are exposed to colorant solutions.

## Conclusions

The findings of the present study showed that preheating of the composite resin is effective in the reduction of color change after long time immersion in coffee solution.
